# Sanitation marketing in Nigeria^☆^

**DOI:** 10.1016/j.worlddev.2025.107022

**Published:** 2025-11

**Authors:** Laura Abramovsky, Nneka Akwunwa, Britta Augsburg, Ephraim Danladi, Erik Harvey, Emmanuel Iorkumbur, Julia Loh, Melanie Lührmann, Abdulazeez Musa, Ada Oko-Williams, Harriet Olorenshaw, Francisco Oteiza, Juan Pablo Rud, Kyla Smith

**Affiliations:** a The Institute for Fiscal Studies, United Kingdom; b ODI, United Kingdom; c Federal Ministry of Water Resources and Sanitation, Nigeria; d World Bank, United States of America; e WaterAid, United Kingdom; f Department of Economics, Royal Holloway,and IFS, United Kingdom; g Frontier Economics, United Kingdom; h Oslo Economics, Norway; i CBM Global Disability Inclusion, Nigeria

## Abstract

We evaluate the effectiveness of a sanitation marketing intervention aimed at increasing the supply of improved toilet products by local businesses and the household demand for toilets in rural Nigeria. Results from a randomized controlled trial show that treated businesses were more likely to produce and market the new toilet model and to engage in the sales of sanitation products more generally. However, we find no discernible impact on household toilet ownership rates. Evidence from interviews with sales agents employed by the business and responsible for about half of the sales suggest that inadequate incentives for sales agents and household affordability constraints may explain these results.

## Highlights

• Sanitation marketing led to a 24% increase in businesses offering sanitation products.
• Despite this increase and marketing efforts, household toilet ownership did not increase.
• Enhancing sales agent incentives and addressing household affordability could improve intervention outcomes.

## 1 Introduction

The global, persistent challenge of ensuring access to safely managed water and sanitation impacts billions of people worldwide, particularly in the Global South. In 2022, around 1 billion people in rural areas still lacked access to basic sanitation, and nearly 400 million practiced open defecation (JMP, 2023). Poor household access to water, sanitation, and hygiene (WASH) has a negative effect on child health and other human capital outcomes (Adukia, 2017, Augsburg and Rodriguez-Lesmes, 2018, Cameron et al., 2022, Orgill-Meyer and Pattanayak, 2020, Sahoo et al., 2015), which in turn hampers overall economic growth (WSP, 2011).

Efforts to achieve Sustainable Development Goal (SDG) 6, which is aimed at ensuring availability and sustainable management of water and sanitation for all, have not been sufficiently effective to meet its 2030 targets (JMP, 2023). Most of the interventions were designed to reduce financial constraints to household investment in sanitation and to induce behavioral change Augsburg, Foster, Johnson, and Lipscomb (2024), even though the vital role of the private sector in improving

the availability of affordable products through sanitation marketing strategies has long been acknowledged.^1^

In this paper, we examine the effectiveness of a private sector intervention increasingly used in rural contexts, referred to as ‘sanitation marketing’, or SanMark^2^. It engages the private sector businesses to improve toilet products offered in local markets and is intended to increase households’ demand for these or functional toilet ownership more generally.^3^

We present new evidence from a field experiment in rural southern Nigeria designed in partnership with governmental and non-governmental organizations (NGOs) that started in 2014. We engaged with a random subset of the private sector through a three-phase intervention: (i) subsidizing the development of a new toilet model tailored to local consumer preferences and undercutting prices of available products^4^; (ii) engaging and training small local businesses to produce and market the model; and (iii) training locals to become sales agents of these toilets in their communities and linking them to the engaged businesses. The business engagement and the demand creation through sales agents were cross-randomized across targeted businesses and communities respectively. In particular, we mapped the universe of target businesses in 245 communities in the states of Ekiti and Enugu. We randomly assigned half of the identified 129 businesses to the training and marketing phase of the sanitation marketing intervention, and half of the 245 communities to the demand creation aspect of the intervention through community engagement. Between 2014 and 2017 we collected four waves of data for businesses and households.

Our findings show that SanMark led businesses to offer new and improved toilets; however, it did not increase toilet ownership among households. We document high levels of awareness and commitment to the newly developed toilet models. More than a year after the business engagement activities, 80% of the treated businesses were aware of the new products and were 14 percentage points (ppts) more likely to include them in their product range; they were also 19 ppts more likely to engage in selling sanitation products in general. Although businesses offered the product and engaged with sales agents mobilized as part of the demand creation activities, our analysis of the household data spanning three years does not show any increase in sanitation ownership due to these new business activities, consistent with the low sales figures reported by businesses and recorded in the administrative sales data of the implementing NGOs.

We consider whether the impact of SanMark on households varied with exposure to another well-known intervention used in rural contexts: Community-Led Total Sanitation (CLTS). It was rolled out as part of the experiment 1.5 years before SanMark was implemented. CLTS is aimed at motivating households to stop practicing open defecation, indirectly sensitizing them to build their own latrines.^5^ We found no differential effect between communities that were previously exposed to CLTS and those that were not.^6^

One reason why we may not find an impact on household ownership of a sanitation facility is the presence of spillovers, which are likely to occur when firms operate in dense local markets. If firms that did not receive SanMark learned from or followed firms that received the intervention, then we should not expect to find differential effects in the supply of the new product and possibly in household adoption. Indeed, we find evidence of information spillovers, as both treated and control groups show similar levels of awareness of the new product post-intervention. However, this awareness does not translate into similar uptake of sanitation in their product line offered. While control businesses are just over 2 ppts more likely to offer sanitation products at endline compared with baseline, the difference is significantly larger for treated businesses at 24 ppts, significant at the 5% level. We show that almost two-thirds of this engagement is due to the adoption of the new toilet model, which only one control business reported offering at endline. Another source of spillover could be if sales agents decide to sell in control communities after training. However, according to our data, about three-quarters of agents’ sales were made in treatment communities. These two findings suggest that while spillovers may have occurred, it is implausible that they can explain the non-impacts.

We conducted additional descriptive analyses of both quantitative and qualitative data to understand the reasons why SanMark may have been ineffective and to provide policy lessons. The analysis reveals two key barriers. For one, the incentive structure for sales agents is suggested to be inappropriate. Despite being responsible for half of all sales, the average agent would have earned less than a fifth of the 2018 legal minimum wage per month, making it unviable as a primary source of income. A redesign of the agent model could generate more sales. Second, according to both agents’ and households’ own reports, the price of the product remains too high for some households and liquidity constraints play a role for others. Addressing financial barriers could enhance the effectiveness of the intervention. One potential approach is to provide subsidies to households, which could reduce the costs they face. This aligns with findings by Hoo et al. (2022), who highlight strong complementarities between SanMark programs and subsidies for toilet construction in rural Cambodia. Additionally, broader literature underscores the critical role of subsidies in alleviating liquidity constraints for sanitation investments across various contexts (Deb et al., 2024, Guiteras et al., 2015, Lipscomb and Schechter, 2018, Pattanayak et al., 2009).^7^ Recent evidence also suggests that making sanitation credit available induces uptake of sanitation products (Augsburg et al., 2023, BenYishay et al., 2017).

More generally, our study contributes to the literature on the opportunities and difficulties of involving the private sector in the provision of sanitation services. For example, Deutschmann, Gars, Houde, Lipscomb, and Schechter (2023) find that prices fall and the use of mechanical de-sludging increases when services are privatized in the context of Senegal, in line with Galiani, Gertler, and Schargrodsky (2005), who show large improvements in health following the privatization of water services in Argentina. However, the reversal of privatization in 2006 suggests that the government’s ability to credibly commit to privatization may be limited Galiani (2021). Augsburg et al. (2023) and BenYishay et al. (2017) show that sanitation microcredit, another market-based approach to sanitation, can increase household ownership of sanitation infrastructure. However, it has also been found that collusive practices among suppliers can increase prices and reduce the uptake of mechanical de-sludging (Houde, Johnson, Lipscomb, & Schechter, 2022), and imperfect contracting between the private and public sectors (Galiani, 2021) has been found to hinder progress in improving the availability of WASH resources and pose limits to its sustainability.

The rest of the paper is structured as follows. Section 2 discusses the context and the intervention. Section 3 describes the research design, Section 4 presents the data, and Section 5 discusses the empirical strategy and results. Section 6 concludes.

## 2 Context and intervention

### 2.1 Context

Nigeria faces a long-standing challenge in eradicating open defecation. According to official statistics, 23% of the population (around 48 million people, most of them rural) were still practicing open defecation in 2021 (FMWR/NBS/UNICEF, 2022).

**Fig. A.1:**
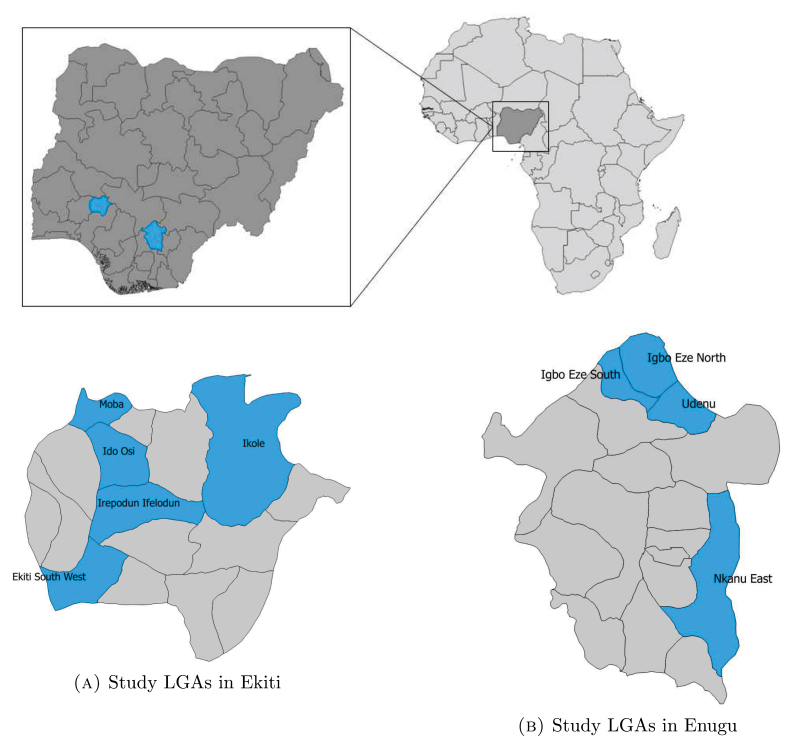
Study location.

As part of the efforts to enhance the understanding of policy effectiveness in addressing challenges in the supply and demand for sanitation, the international NGO WaterAid launched the Sustainable Total Sanitation (STS) program in 2013, working with local government authorities (LGAs)^8^ and local NGOs, and included a research component (Abramovsky et al., 2023, WaterAid, 2018). The research was implemented in two study states, Ekiti and Enugu. (See Fig. A.1 in Appendix A for a map of study areas.) Both states displayed low pre-intervention rates of access to improved sanitation, which resulted in high rates of open defecation.^9^

STS consisted of two main interventions. The first one, CLTS, mentioned in the introduction, was implemented in the first half of 2015 and aimed at empowering communities to eradicate open defecation. Abramovsky et al. (2023) showed that CLTS reduced open defecation and increased the adoption of latrines in the poorest treated communities relative to control communities in these two states.

The second intervention, and the subject of this paper, is SanMark. It targeted local businesses to produce and market newly developed, affordable, and improved toilet products. The engagement of businesses happened 1.5 years after communities were exposed to the CLTS intervention. As we describe below, SanMark was implemented both in communities that received CLTS and in communities that did not. This allows us to examine whether SanMark and CLTS interact.

### 2.2 Intervention

The SanMark intervention developed new toilet models based on research of local consumer preferences and targeted specific businesses to encourage them to produce and sell these new models to rural households, including by recruiting and using sales agents.

**Fig. B.1:**
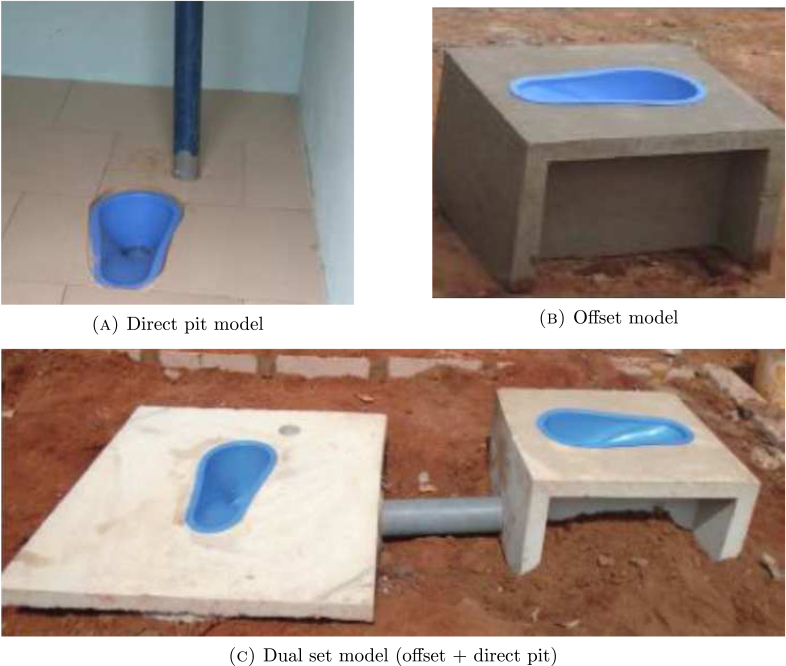
WET models.

The first phase of the SanMark intervention consisted in product design and testing of the Water Easy Toilet (WET) models in 2013. These designs were developed to deliver more affordable, safer, and less water-intensive improved toilets. The design process, as described in Abramovsky, Augsburg, and Oteiza (2019), involved hiring and supporting a private product designer who conducted three weeks of qualitative field research (or ‘Deep Dive’) to understand consumer preferences and commercial supply chains for rural sanitation in the intervention areas. Building on insights gained, the team undertook an iterative human-centered design approach to develop products and business models. The ultimate goal was to design a small suite of affordable, understandable, and desirable toilet products that addressed the key concerns from the previous Deep Dive. Three rounds of iterative development and testing were conducted before settling on the final product. Three new WET models were invented: a direct pit toilet, an offset toilet (which includes an additional height offset), and a dual set that combines both while sharing a single pit. These are shown in Appendix Fig. B.1. WET dual set toilets cost ₦28,500 (US$174 at 2014 prices), while the WET direct pit model cost ₦21,000 (US$128), and the offset model cost ₦7375 (US$45) on average. These are substantially lower amounts than the median cost that households reported in the baseline survey in 2014, conducted as part of the STS research, at ₦35,000 (US$214)

The second phase of the SanMark intervention focused on business engagement. It was aimed at preparing businesses to produce these new WET designs and to sell the products to households, mainly in nearby rural communities. Participating businesses were invited to product development sessions that discussed the advantages of WET models and described the steps for construction and installation, providing hands-on training during which participants learned to construct WET models. Next, businesses were invited to training sessions with business development consultants, teaching basic management skills and data handling to understand demand in surrounding communities. These were followed by regular visits from staff of the international NGO for support regarding basic business problems.

**Fig. B.2:**
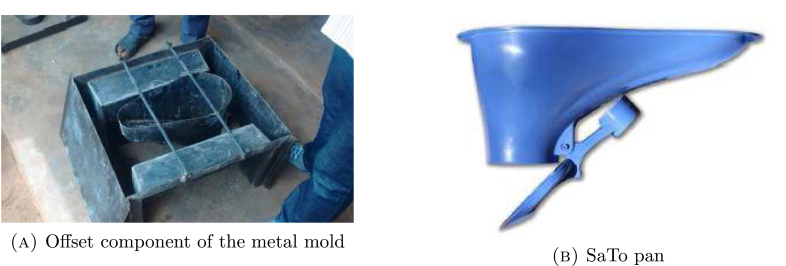
Components of WET models.

After participating in these training sessions, SanMark businesses were provided with product support, which included free access to the metal mold for the casting of components. In this last part of the business engagement phase, suppliers would borrow these molds free of charge from the international NGO; otherwise they would cost an estimated US$400 to acquire. In addition, only SanMark businesses were able to purchase pre-fabricated plastic SaTo pans,^10^ a key component of the WET models, at a cost of around ₦1000, about US$3. At the time of the intervention, these pans were not produced in Nigeria yet, and the international NGO was the only importer of the plastic pans into the country. The mold and pan are displayed in Appendix Fig. B.2.

The third and final phase of the SanMark intervention comprised community engagement aimed at stimulating households’ demand for and sales of WET models. Sales agents were recruited from randomly selected communities by staff from LGAs’ WASH units. Individuals recruited as sales agents were invited to attend training sessions to learn about the product line, pricing, and pitching techniques. These agents were tasked with door-to-door sales of WET products (WaterAid, 2018). WASH unit staff gave each sales agent a list of SanMark businesses that offered WET products. They could approach households in any community, not only in the communities they resided in. All compensation earned by sales agents came from making sales, and the commissions were to be negotiated directly with participating business owners, without influence from the international NGO. In addition, community-level marketing activities aimed at promoting the health benefits and aspirational effects of owning a WET model among households were organized.^11^

**Fig. 1:**
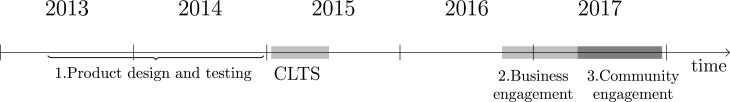
STS program timeline. Notes: The figure indicates the timline of STS intervention implementation. The three SanMark phases are denoted with numbers.

Fig. 1 summarizes the STS project timeline and the sequence of activities. The first phase of the SanMark intervention began in September 2013 with market research and the development of the design of the WET models. Shortly after, and independently of SanMark’s product development, CLTS was implemented, as described in Abramovsky et al. (2023). Phase two, which focused on business engagement, started 3 years from initiation (and around 1.5 years after CLTS was implemented), in September 2016. The third phase, community engagement, began 6 months later in February 2017, with the recruitment of sales agents. Community-level marketing events and sales agents’ door-to-door activities were then implemented between March 2017 and October 2017.

## 3 Research design

The research design is a two-level randomized controlled trial. First, businesses are randomized, allowing for an assessment of whether and the degree to which the private sector becomes involved in the sanitation market and sells new sanitation products (such as the WET product range) to households. Second, communities are randomized to assess impacts on household toilet ownership. The two SanMark randomization exercises were run independently of each other. This means that a SanMark *business* may be located in either a SanMark or control *community*.

### 3.1 Business randomization

We conducted a census of targeted businesses between April and May 2014 and identified 135 eligible businesses in the study areas. These businesses were primarily concrete block manufacturers and merchants, due to the heavy concrete component in the WET design.

The intervention was piloted with 6 of the 135 businesses in Igbo Eze North LGA in early 2016. The remaining 129 businesses in Ekiti and Enugu were randomly assigned to either the SanMark treatment group or the control group, stratified at the LGA level. Treated businesses were given access to WET construction workshops, business training, and the metal molds and plastic pans needed to make the WET products.

Our primary outcomes are product awareness and adoption (of new WET models), as well as engagement in selling sanitation products in general.

**Fig. C.1:**
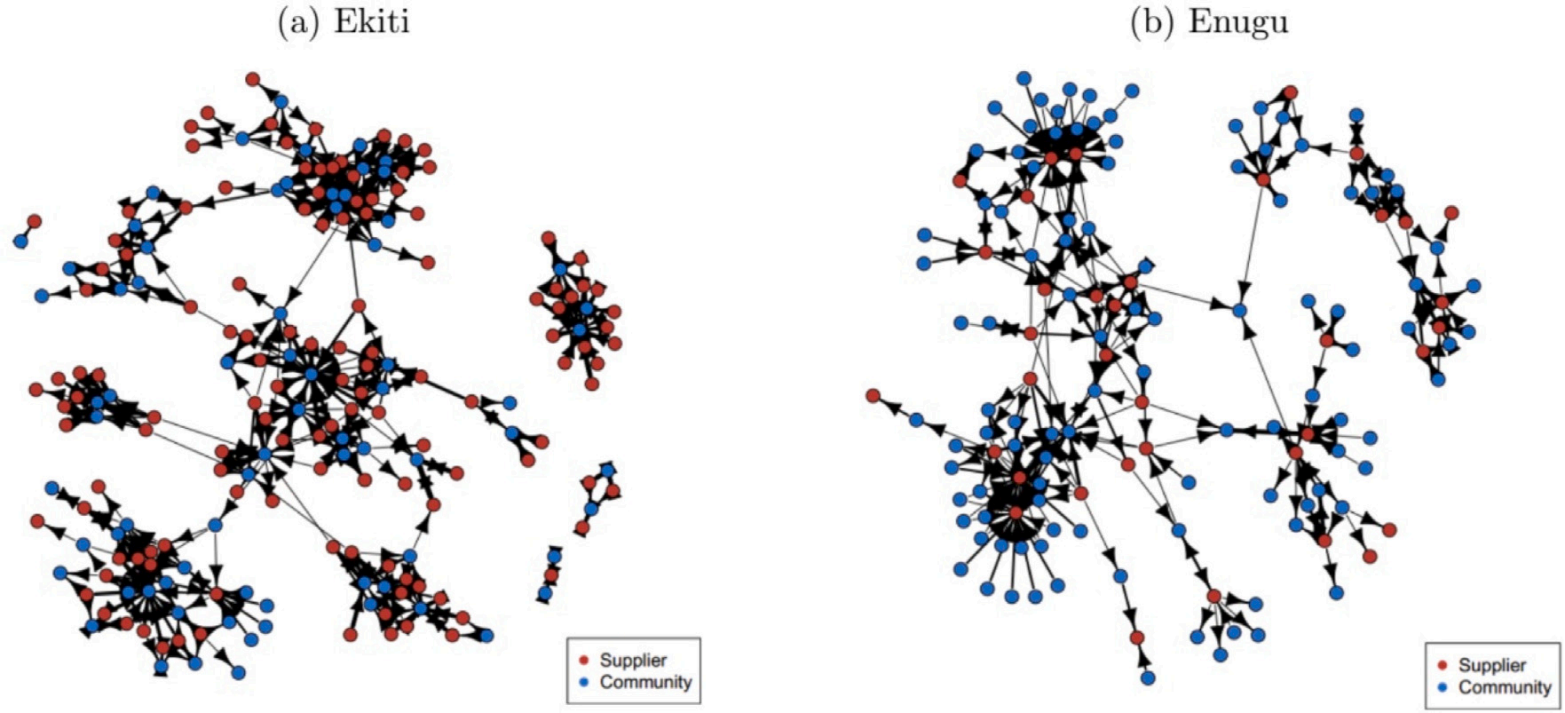
Cement block transaction network diagram.

Such engagement could in turn affect business performance through two channels. First, business training could improve business acumen, which may have a direct impact on the way businesses are run, regardless of whether or not they decide to become involved in sanitation. Second, involvement in the sale of sanitation products could have a direct impact on business profits. Of course, involvement in sanitation could also divert attention from other profit-generating activities, making the overall impact on firm performance ambiguous. Unfortunately, providing empirical evidence on whether and how SanMark affects business performance is beyond the scope of this paper, for two reasons. First, measuring the profits of micro-enterprises is extremely difficult and often results in data with significant measurement error (de Mel, McKenzie, & Woodruff, 2009). Despite our efforts to collect this data, it proved extremely difficult to obtain accurate information. Second, as shown in Appendix Fig. C.1, the targeted businesses operate in highly interconnected local markets. Our random assignment of businesses to treatment and control groups within these markets allows for potential spillover effects from treatment to control. For example, treatment businesses may have gained a competitive advantage, potentially causing control businesses to suffer, or control businesses may have changed their activities in response and improved their performance. A simple comparison of business performance between treatment and control would obscure such potential externalities.^12^ We will provide some limited evidence on these outcomes, bearing in mind these two constraints.

The possibility of spillovers is not only relevant for business performance outcomes. Estimates of technology awareness and adoption may also be biased. Although we show that awareness of the new product is high among both treated and control businesses, the limited availability of metal molds and SaTo pans, which are essential for the construction of WET models, constrains businesses in terms of product and technology adoption. These resources were provided directly to the treated enterprises by the international NGO and would have been difficult for the control businesses to obtain or replicate. To measure the extent to which this might have happened, we also asked control businesses about potential adoption and find that only one business claims to have adopted WET technology. Furthermore, we consider the adoption of sanitation products in general as a key outcome and show that treated businesses are significantly more likely than controls to be involved in this market. We therefore believe that we are well placed to examine the impact of the intervention on our primary outcomes.

### 3.2 Community randomization

To evaluate the effectiveness of sales agents and community-level marketing activities in increasing household ownership of improved toilets, the second level of randomization was clustered at the community level. A community in this study refers to ‘triggerable units’ (TUs), which are groups of geographically close and socially interconnected villages, neighborhoods, or quarters as defined on the basis of implementers’ previous experience of working in these areas (see Abramovsky et al., 2023 for further details). The sample consists of 293 TUs, or clusters, after removing 30 communities in Igbo Eze North where the intervention was piloted. This cluster randomization allows us to identify the causal impact of the door-to-door agents and community activities on household toilet ownership, assuming no spillovers from treated to control TUs. To minimize such potential spillovers, “buffer” areas were introduced around TUs to ensure that no two clusters were in close geographical proximity. In addition, households within a treated cluster were targeted by recruiting sales agents who resided in these communities. We note, however, that the activities of the sales agents were not limited to SanMark treated TUs. As a result, any impacts found by comparing households in treatment and control TUs can be interpreted as a lower bound.

As mentioned above, communities in the study may also have received CLTS in the past. This would have been either as part of or prior to the initial STS intervention. An important aspect of the community-level randomization for SanMark is that the TUs were randomly assigned to treatment and control groups regardless of their CLTS treatment status, but stratified by CLTS treatment, distinguishing whether a community had received CLTS before (‘Pre-CLTS’) or as part of the STS program (‘CLTS’). This ensured an even distribution of the six cross-treatment types across the LGAs, with the aggregated figures shown in Table 1.

**Table 1:** Number of communities by treatment status.

	SanMark		Control		Total	
	Freq.	%	Freq.	%	Freq.	%
Pre-CLTS	37	26.4	41	26.8	78	26.6
CLTS	52	37.1	57	37.3	109	37.2
Control	51	36.4	55	36.0	106	36.2

Total	140	100	153	100	293	100

### 3.3 Data collection

To obtain information on both businesses and households, we collected a considerable amount of primary data over the four years of the study.

On the business side, we started in 2014 with a mapping of all businesses that could be eligible for the SanMark intervention. Those identified as eligible, 135 in total, were then interviewed four times subsequently. The first round of interviews, wave 1, took place in December 2014 and early January 2015. Wave 2 took place between December 2015 and February 2016 and serves as the baseline data for the SanMark evaluation. Wave 3 took place in March–April 2017, approximately six months after the engagement of businesses and just before the engagement of sales agents and communities. Wave 4 (also referred to as the endline survey) was collected in October 2017. At each of the four survey waves, we collected detailed information on owner characteristics and personality, as well as information on business characteristics, including sales, costs, and sanitation-related products offered. At wave 4, in addition to the business survey, data were also collected from sales agents, including the number and location of WET sales and the amount of commission received.

To evaluate impacts at the household level, we used a household panel initiated for the CLTS evaluation. We started with a census conducted in mid-2014, which provided the sampling frame for households. A total of 50,333 households (27,888 from Enugu and 22,445 from Ekiti) were interviewed to collect basic household information. From each TU (or cluster), 20 households were randomly selected, resulting in a final study sample of 5639 households and 293 cluster areas, evenly distributed between Ekiti and Enugu and representing just over 11% of the sampling frame. Households were interviewed in parallel with businesses during waves 1–4. The survey collected information on household composition, demographics, assets, consumption, and importantly, investment in and ownership of sanitation infrastructure.

## 4 Data

We use the rich data collected to provide contextual information about our study businesses and households, and to test whether the randomizations were successful in creating observationally equivalent groups.

### 4.1 Businesses

Panel A of Table 2 presents the summary statistics for study businesses. We show the baseline mean and standard deviation for the control group (in the post-attrition sample), the difference in means between the treatment and control groups, and the *p* value for the *t* test of equality of these means.^13^

The businesses, which had on average 4 full-time employees, had typically been established 6 years prior to the baseline survey. More than 70% were formally registered. A large majority had owners with at least secondary education (85%). For just over 65% of the businesses, households were the main customer base in terms of the number of products sold (as opposed to artisans, contractors, NGOs, or government), and about 80% of customers would come to the business location to pick up products. In line with the dense networks depicted in Fig. C.1, the average business has 7 other similar businesses within a 5 km radius. Almost 7% of businesses were offering some sanitation products at baseline. Almost none of the businesses were connected to a power grid, but just over one-third owned an electricity generator. Connection to an improved water source was also negligible at just under 3%, as was access to an internet connection (1.45%). Most businesses (71%) used mobile phones, and just over 60% of businesses had reliable means of transport.

In terms of business performance, the reported typical monthly profit is ₦147,000 (about US$408 at the time of the study). More than half of the enterprises (58%) reported that their underlying turnover had increased in the past year, and 40% of the businesses reported that they had taken out a loan in the past.

The treatment–control comparison in the last columns of the table shows that there are no significant differences between control and treatment businesses at baseline, except for a lower proportion of treatment businesses reporting owners with no educational qualifications. This difference of 4.76 ppts is significant at the 10% level. Treatment businesses also had, on average, one more full-time employee than control businesses—but this difference is not statistically significant. Joint tests of these baseline characteristics on treatment status yield an F-statistic of 1.3 and a corresponding *p*-value of 0.20, suggesting that these characteristics do not jointly predict the likelihood of receiving treatment.

Panel B of Table 2 shows an attrition rate of 6% among control businesses, defined as being closed in both post-treatment survey waves. An additional 4% of control businesses do not provide data on post-treatment business performance outcomes, which means that no information on these outcomes is available for 10% of control businesses. As indicated in the last column of the table, these attrition/non-reporting rates are not statistically different among treatment and control businesses. In Appendix Table E.2, we further regress these attrition indicators on treatment status and business characteristics. Even after controlling for characteristics, treatment does not predict attrition.

**Table 2:** Balance in baseline characteristics between treatment and control businesses.

	All	Control			Treatment-Control	
	Obs.	Obs.	Mean	SD	Coeff.	*p*-value
**Panel A - Business summary statistics**						
Years in Existence	101	54	6.17	5.43	0.60	0.63
Number of Full-time Employees	117	65	4.28	3.57	1.22	0.11
Registered with authorities (%)	117	65	72.31	45.10	−1.15	0.89
Level of education of main owner						
No qualifications (%)	114	63	4.76	21.47	−4.76	0.08*
Primary education (%)	114	63	17.46	38.27	−3.73	0.58
Secondary education (%)	114	63	41.27	49.63	3.83	0.68
Tertiary education (%)	114	63	36.51	48.53	4.67	0.61
Households main customers (%)	117	65	64.62	48.19	12.31	0.14
Customers pickup products (%)	117	65	81.54	39.10	−2.69	0.72
Innovation Score	117	65	4.02	3.65	−0.50	0.43
Number of other CBP businesses within 5 km	117	65	6.91	3.36	−0.31	0.62
Involved in sanitation (%)	117	65	6.15	24.22	−4.23	0.24
*Infrastructure*						
Connected to power grid (%)	117	65	3.08	17.40	4.62	0.28
Owns electricity generator (%)	117	65	40.00	49.37	2.31	0.80
Improved water source (%)	117	65	3.08	17.40	−3.08	0.16
Business has internet connection (%)	117	65	3.08	17.40	0.77	0.82
Business uses cell phones (%)	117	65	69.23	46.51	−5.77	0.51
Have reliable means of transport (own) (%)	117	65	63.08	48.64	10.00	0.25
*Business performance*						
Typical real monthly profits (’000 ₦, 2009 prices)	117	65	146.78	535.35	−93.10	0.28
Ever received a loan (%)	117	65	36.92	48.64	−2.31	0.80
Sales increased in past year (%)	117	65	58.46	49.66	−4.62	0.62
**Panel B - Attrition**						
Closed in both post-treatment survey waves	129	69	0.06	0.24	0.08	0.15
Missing business performance outcomes	129	69	0.10	0.30	0.08	0.19

*Note*: Data from baseline and second wave business surveys carried out before the intervention. If business is not surveyed in the baseline survey, data from the second wave survey is used. The availability of the business for the baseline survey is not correlated to treatment status. The variation in sample sizes across variables is due to missing responses for individual questions in the survey. Variables that fed into the innovation score are displayed in Appendix Table D.1. * p<0.10, ** p<0.05, *** p<0.01. Obs. stands for observations, SD for standard deviation, coeff. for coefficient.

### 4.2 Households

Table 3 provides information on households. Panel A focuses on household characteristics at baseline. A typical household is headed by a male (64%), aged 55 years, who has completed primary school (68%) and is employed (77%). Households have on average four members, and 30% have at least one child under the age of 6 years. Agriculture is the main activity of almost half (48%) of the households. In terms of ownership of sanitation infrastructure, an average of 36% of control households report owning a working toilet of any type, while a slightly smaller proportion (33%) report owning a working improved toilet.

Comparing treatment and control households, we find that a higher proportion of treated household heads are employed. This difference of 2.8 ppts is significant at the 10% level. All other differences in household characteristics are not significant at the 10% level.

Panel C provides information on the location of households. They are located on average 7 km from the LGA headquarters, and a typical household has just under 1500 households in its vicinity (5 km radius).

We conducted F-tests to investigate the joint significance of these baseline characteristics in Panels A, B, and C on the probability of a household receiving treatment. The resulting F-statistic of 1.25 with a corresponding *p*-value of 0.25 suggests that these characteristics do not jointly predict the probability of a household being treated.

As for businesses, we present two indicators of household attrition, shown in Panel D. The first indicator is 1 if a household was not surveyed at endline, and 0 otherwise; the second indicator includes as 1 those households for which data on sanitation ownership are missing at endline. Some 14% of households in the control group are not surveyed at endline, and 15% have missing and/or have incomplete data on sanitation ownership. These attrition indicators do not differ significantly between treatment and control. In Appendix Table E.3 we show that treatment status does not predict attrition, with and without controlling for observable baseline characteristics.

**Table 3:** Balance in baseline characteristics between treatment and control households .

	All	Control			Treatment-Control	
	Obs.	Obs.	Mean	SD	Coeff.	*p*-value
**Panel A - Household Characteristics**						
HH head male (%)	4703	2453	63.80	48.07	0.96	0.59
HH head age (years)	4703	2453	55.24	17.39	0.41	0.54
HH head employed (%)	4703	2453	76.84	42.19	2.80	0.09*
HH head finished primary school (%)	4703	2453	68.24	46.56	−1.93	0.33
Household size	4703	2453	4.10	2.38	−0.01	0.90
Household has at least 1 child below 6 y/o (%)	4703	2453	30.82	46.18	0.16	0.93
HH primary activity is farming (%)	4703	2453	47.74	49.96	4.66	0.19
Relative asset wealth index score (mean = 0, SD = 2)	4703	2453	0.04	2.18	0.05	0.72
**Panel B - Toilet Ownership**						
Own a functioning toilet (any type) (%)	4703	2453	36.24	48.08	−1.00	0.74
Own a functioning, improved toilet (%)	4703	2453	33.31	47.14	−1.57	0.59
**Panel C - Community Characteristics**						
Distance to nearest LGA HQ (in km)	291	152	7.26	4.42	−0.35	0.52
Number of households within a 5km radius	291	152	1,497	1,025	−0.81	0.99
**Panel D - Attrition**						
Not surveyed at endline (%)	5639	2955	14.08	34.79	−1.56	0.21
Missing data on sanitation ownership (%)	5639	2955	14.79	35.50	−1.60	0.21

*Note*: Data from community survey during wave 1 data collection and wave 2 household survey carried out before the intervention. Variables that fed into the asset wealth index are displayed in Appendix Table D.1. * p<0.10, ** p<0.05, *** p<0.01. Obs. stands for observations, SD for standard deviation, Coeff. for coefficient, HQ for head quarter, and BL for baseline.

## 5 Results

We first discuss the impact of SanMark on businesses, namely on awareness and adoption of the new toilet technology, and more generally on participation in sanitation. We then look at the impact of SanMark on household ownership of toilets, exploring whether this effect varies between communities with and without a SanMark target business. We also examine whether the impact of SanMark on toilet ownership varies across communities depending on whether or not they were previously exposed to CLTS.

**Table 4:** Firm awareness and offering of WET products and sanitation by SanMark treatment status.

	Firm knows about WET products	Firm offers WET products to consumers	Firm is involved in sanitation
	(1)	(2)	(3)
SanMark	0.09	0.15***	0.24***
	(0.06)	(0.06)	(0.07)

Control mean at endline	0.85	0.03	0.08
No. of obs.	117	117	117

*Note:* Estimates based on OLS regressions using equation (1) with controls. *p*-values from individual testing are presented in brackets. All specifications include strata indicators (LGAs). Standard errors are clustered at the business level and are shown in parentheses. * p<0.10, ** p<0.05, *** p<0.01. Dependent variable by columns: (1) *Firm knows about WET products*: an indicator variable equal to 1 if the business owner reports knowing about the WET product, 0 otherwise; (2) *Firm offers WET products to customers*: indicator variable equal to 1 if business owner reports offering WET products, 0 otherwise; (3) *Firm involved in sanitation:* indicator variable equal to 1 if business reports to offer WET products (as in Column 2) or having positive revenues from selling alternative toilet models, 0 otherwise.

### 5.1 Impact of SanMark on business activities

To estimate impacts of SanMark on business outcomes, we use the following specification:

(1) $$y_{i,g}=\alpha +\nu SanMark_{i}^{b}+X_{i}^{\prime }\beta +\mu_{g}+\epsilon_{i,g}$$

where $y_{i,g}$ is the outcome of interest of business i in LGA g, $SanMark_{i}^{b}$ is an indicator variable equal to 1 if business i was selected into the SanMark program and 0 otherwise. Our coefficient of interest is ν, which represents the difference in outcomes for the SanMark businesses before and after the intervention. $X_{i}^{\prime }$ is a vector of baseline control variables at the business level. Finally, $\mu_{g}$ controls for time-invariant differences in the outcomes across LGAs, given that our randomization was stratified at this level.

The randomized nature of the intervention combined with the finding that randomization was successful in creating observationally equivalent groups (Table 2) provides evidence that supports the interpretation of ν as the causal effect of the interventions. We further show that the results are robust to alternative specifications, namely adding controls and using an ANCOVA specification, where information on the outcome pre-treatment is available:

(2) $$y_{i,g,Post}=\alpha +\nu SanMark_{i}^{b}+\theta y_{i,g,Pre}+X_{i}^{\prime }\beta +\mu_{g}+\epsilon_{i,g,Pre}$$

where we pool information from waves 3 and 4 into the post-intervention outcome. Here, $y_{i,g,Post}$ represents the post-intervention outcome of interest for business i in LGA g post-intervention, and $y_{i,g,Pre}$ represents the post-intervention outcome of interest for business i in LGA g before the intervention. $X_{i}^{\prime }$ is a vector of control variables at the business level, while $\delta_{t}$ allows for a common time trend.

We focus on intention-to-treat (ITT) coefficients by estimating the effect of being approached by the international NGO to participate in SanMark’s business activities.

Table 4 shows our impacts on business-related primary outcomes using the sample of businesses that remain open post-treatment.^14^ We find that at the time of wave 4 (late 2017), the general awareness of the WET product line among treatment businesses was high, with 85% confirming that they were aware of the WET products. This suggests that the intervention was successful in introducing the product line to businesses. The increase in awareness was, however, also observed among firms that did not receive the intervention, as 80% of control businesses reported knowing about the new toilet models. We therefore do not find a statistically significant difference between SanMark and non-SanMark firms in terms of product awareness (column 1).

Awareness translates into adoption. Column 2 of Table 4 shows that SanMark firms were significantly more likely to offer these products, with 18% of SanMark businesses surveyed reporting that they offered them to their customers. In column 3 we look at general involvement in sanitation, defined as offering WET products (as in column 2) or having positive revenues from selling alternative toilet models. We see a significant impact of 24 ppts on this outcome. This is an encouraging result, as it suggests that SanMark has the potential to develop and expand the sanitation market.

It should be noted that 3% of the control enterprises also reported offering WET products (column 2). It is likely that this is actual contamination (rather than reporting error), because these businesses also reported having been offered and attending business training by the international NGO. One business even reported receiving the mold. Although small, this contamination means that the impact estimates presented are lower bounds. Importantly, we see little evidence of uptake of sanitation products in general by control enterprises. Their commitment to sanitation increases from 5.7% at baseline to 8% at endline. The impact estimates are robust to the addition of controls (Appendix Table F.1) and to the use of an ANCOVA specification for the outcome of whether the business is engaged in sanitation (Appendix Table F.2).

We next analyze how this increase in offering translates into actual sales of WET models. To do this, we use two data sources: (i) internal administrative program data collected by the international NGO and (ii) primary business survey data on reported sales of WET units.

**Fig. 2:**
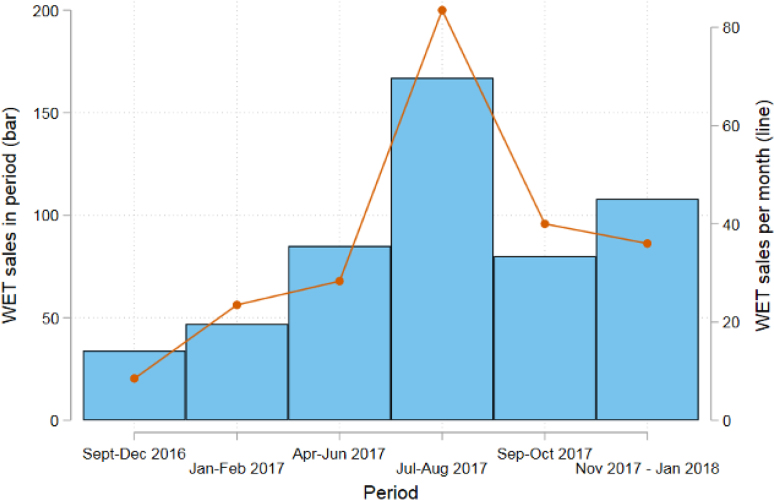
WET sales (of any kind) during the period of study.

The NGO administrative data on WET sales (Fig. 2) show a modest total number of WET sales throughout the study period. In the first four months of the intervention, fewer than 50 units were sold, which equates to about 20 units per month. We observe a slow improvement until April 2017, followed by an increase and peak in sales in July–August 2017, when 200 units were sold. This coincides with the introduction of phase 3 of SanMark, including door-to-door sales agent activities, which will be discussed in more detail in Section 5.2. Consistent with the low figures observed in the administrative data, the treated businesses reported selling an average of 14 WET units per month (the 3% of control businesses reported selling an average of 6 units per month). Businesses reported a sevenfold increase in monthly WET sales between wave 3 (April–May 2017) and wave 4 (late 2017). We note that the decline in the pace of WET sales observed from September 2017 should not necessarily be a cause for concern, as it could be due to the seasonality of household investment in toilets. There is no longer-term data available to explore this further. We discuss the potential impact on business profits in Appendix G, which suggests no differences between treatment and control business performance at endline. Although these results are consistent with the small number of sales, we consider these findings to be indicative only, given the concerns about measurement error and potential spillovers discussed above.

The evidence supports the idea that the introduction of door-to-door sales agents, who marketed WET models directly to households, played a role in increasing sales of WET models. In a survey of a subset of businesses, we find that 41% had contact with these sales agents, and of these, 62% reported making new sales as a result of the agents’ work.

WaterAid Nigeria internal tracking data (line); business surveys (bars)

### 5.2 Impact of SanMark on household toilet ownership

To estimate the impact of SanMark on the rate of improved toilet ownership, we use a difference-in-differences design, akin to Eq. (3):

(3) $$y_{i,c,t}=\alpha +\nu SanMark_{c}+\gamma SanMark_{c}\times Post_{t}+X_{i}^{\prime }\beta +Post_{t}+\omega_{g}+\epsilon_{i,c,t}$$

where $y_{i,c,t}$ represents the outcome of interest for household i in cluster c in period t. $Post_{t}$ is an indicator equal to 1 if the period of analysis t is after the introduction of SanMark (in waves 3 and 4) and 0 otherwise (in wave 2). $X_{i}^{\prime }$ is a vector of control variables at the household level, while $Post_{t}$ allows for a common time trend. We include LGA fixed effects, $\omega_{g}$, to account for unobserved area effects and given stratification. Community-level SanMark treatment status is defined as $SanMark_{c}$, and the parameter γ captures the average ITT effect of SanMark on household improved toilet ownership.

Table 5 reports the results for households reporting on sanitation ownership pre- and post-treatment.^15^ The first dependent variable is a dummy equal to 1 if the household owns any functioning toilet, and 0 otherwise, and the second outcome variable is a dummy if the functioning toilet is an improved one. We find no evidence of an effect of the intervention on functioning and improved toilet ownership (coefficients of −0.01 and −0.02 respectively), suggesting that the intervention did not produce a marked shift in sanitation ownership. Results are similar when accounting for pre-intervention covariates and using an ANCOVA specification, as shown in Appendix Table H.1.^16^

**Table 5:** Impact of SanMark on toilet ownership.

	HH owns a functioning toilet	HH owns a functioning improved toilet
	(1)	(2)
SanMark	−0.01	−0.02
	(0.02)	(0.02)
SanMark × Post	−0.01	−0.02
	(0.02)	(0.02)
Control mean at endline	0.54	0.49
No. of communities	291	291
No. of HHs	4,703	4,703

*Note:* Estimates based on OLS regressions using equation (4). All specifications include strata indicators (LGAs). Standard errors are clustered at the community level and are shown in parentheses. * p<0.10, ** p<0.05, *** p<0.01. Dependent variable by columns: (1) *HH owns functioning toilet*: an indicator variable equal to 1 if the household reports to own a functioning toilet, 0 otherwise; (2) *HH owns a functioning improved toilet*: indicator variable equal to 1 if household (HH) reports to own a functioning toilet that is improved as defined in WHO/UNICEF (2015).

#### 5.2.1 Heterogeneous impact by prior exposure to CLTS

Practitioners believe that a combination of SanMark with CLTS could have stronger joint impacts than they can achieve separately. In particular, demand has to be stimulated via CLTS before implementing SanMark. Our research design provides us with the opportunity to test this hypothesis because CLTS was implemented in these communities prior to SanMark. The equation below enables the identification of heterogeneous impacts of SanMark by CLTS treatment status:

(4) $$y_{i,c,t}=\tau_{0}+\tau_{1}S_{c}+\tau_{2}S_{c}\times Post_{t}+\tau_{3}S_{c}\times CLTS_{c}\times Post_{t}+\tau_{4}CLTS_{c}+\tau_{5}S_{c}\times CLTS_{c}+\tau_{6}CLTS_{c}\times Post_{t}+Post_{t}+X_{i,c,t}^{\prime }\beta +\omega_{g}+\epsilon_{i,c,t}$$

where variables are defined as previously, and $CLTS_{c}$ is community-level CLTS treatment status. This difference is identified by $\tau_{3}$. A CLTS indicator variable is included, as well as its interaction with the time dummy that accounts for the period after SanMark was implemented. Results are presented in Table 6. Results using alternative specifications are presented in Appendix Table H.3. Note that in this specification we include both types of CLTS communities (those that received CLTS before the STS intervention and those that received CLTS as part of the STS intervention), implying that CLTS is not exogenous. In Appendix Table H.2, we exclude pre-CLTS communities and keep only those communities that were randomly allocated CLTS (or not), implying that all coefficients can be interpreted as causal. Results are consistent across both samples and using alternative specifications (Appendix Table H.4).

Differences in ownership of functioning and improved toilets are quantitatively and statistically insignificant between SanMark households triggered by the CLTS interventions and other SanMark households. Initially, it was planned that SanMark would be rolled out straight after the CLTS intervention, thereby providing an affordable and safe improved toilet option to meet any increased demand as a result of the CLTS intervention. However, delays led to a gap of 15 months before the start of SanMark, and households triggered by CLTS to construct a toilet did so shortly after the intervention implementation (Abramovsky et al., 2023), potentially explaining non-impacts in the interaction.

**Table 6:** Impact of SanMark on toilet ownership by CLTS treatment group.

	HH owns a functioning toilet	HH owns a functioning improved toilet
SanMark × Post ($\tau_{2}$)	−0.04	−0.05
	(0.04)	(0.03)
SanMark × Post × CLTS-treated ($\tau_{3}$)	0.04	0.05
	(0.05)	(0.04)
Joint effect for SanMark and CLTS-treated ($\tau_{2}+\tau_{3}$)	0.00	0.00
	(0.03)	(0.03)

Control mean at endline	0.54	0.49
No. of communities	291	291
No. of HHs	4,703	4,703

*Note:* Estimates based on DiD specification using equation (5). All specifications include strata indicators (LGAs). Standard errors are clustered at the community level and are shown in parentheses * p<0.10, ** p<0.05, *** p<0.01. Dependent variable by columns: (1) *HH owns functioning toilet*: an indicator variable equal to 1 if the household reports to own a functioning toilet, 0 otherwise; (2) *HH owns a functioning improved toilet*: indicator variable equal to 1 if household (HH) reports to own a functioning toilet that is improved as defined in WHO/UNICEF (2015).

#### 5.2.2 Interaction of business training and community marketing

We next turn to analyzing the impacts of SanMark on toilet ownership differences between communities with or without a SanMark-treated businesses. We estimate heterogeneous impacts using Eq. (4) interacting $S_{c}$ with whether a treated business was located within the community. For this analysis, we exclude control communities served by a treated supplier to specifically compare treated communities containing a treated supplier with control communities lacking such a supplier.^17^

We find that households that live in SanMark communities in which a SanMark-treated business is located do indeed have a higher impact on toilet ownership than households residing in communities without a treated business, where the estimated coefficient is negative at 0.04, shown in Table 7). Results are robust to alternative specifications (Appendix Table H.6, Table H.7), including a specification where we do not drop any observations and instead interact $SanMark_{c}$ with the number of SanMark suppliers within a 1 km radius, and within a 5 km radius. We find no heterogeneous impacts with this intensity measure. The coefficient is basically zero and insignificant.). However, neither impact is statistically significant, nor is the difference between them statistically significant.

**Table 7:** Impact of SanMark on toilet ownership by treatment intensity.

	HH owns a functioning toilet	HH owns a functioning improved toilet
SanMark × Post	−0.04	−0.04
	(0.03)	(0.03)
SanMark × Post × Treated main supplier	0.08	0.05
	(0.05)	(0.05)

Control mean at endline	0.56	0.53
No. of communities	249	249
No. of HHs	4,022	4,022

*Note:* Estimates based on DiD specification using equation (5). All specifications include strata indicators (LGAs). Standard errors are clustered at the community level and are shown in parentheses. * p<0.10, ** p<0.05, *** p<0.01. Dependent variable by columns: (1) *HH owns functioning toilet*: an indicator variable equal to 1 if the household reports to own a functioning toilet, 0 otherwise; (2) *HH owns a functioning improved toilet*: indicator variable equal to 1 if household (HH) reports to own a functioning toilet that is improved as defined in WHO/UNICEF (2015). Households with both control and treated main suppliers are dropped.

### 5.3 Sales agents: training, sales, and earnings

We finally examine the role of sales agents’ activities in household sanitation uptake. In particular, we consider agents’ reports on location and number of visits, number of sales, and commission earned. These agents were interviewed in the endline survey.

Almost all agents confirmed having attended the training sessions. However, only 48% were still active about half a year after the training, defined as having attempted at least one sale during the last month. Some 30% of agents report having been involved in a successful sale, for which they received an average commission of US$3.30 from businesses. Based on reported working hours (on average of 2.5 days a week and for 3 h per day), the average active agent would have earned a monthly wage of US$8 through toilet sales—less than a fifth of the 2018 legal minimum wage of US$60 a month. Consistent with this, most (95%) active agents do not rely on making WET sales as their main source of income.

**Fig. J.1:**
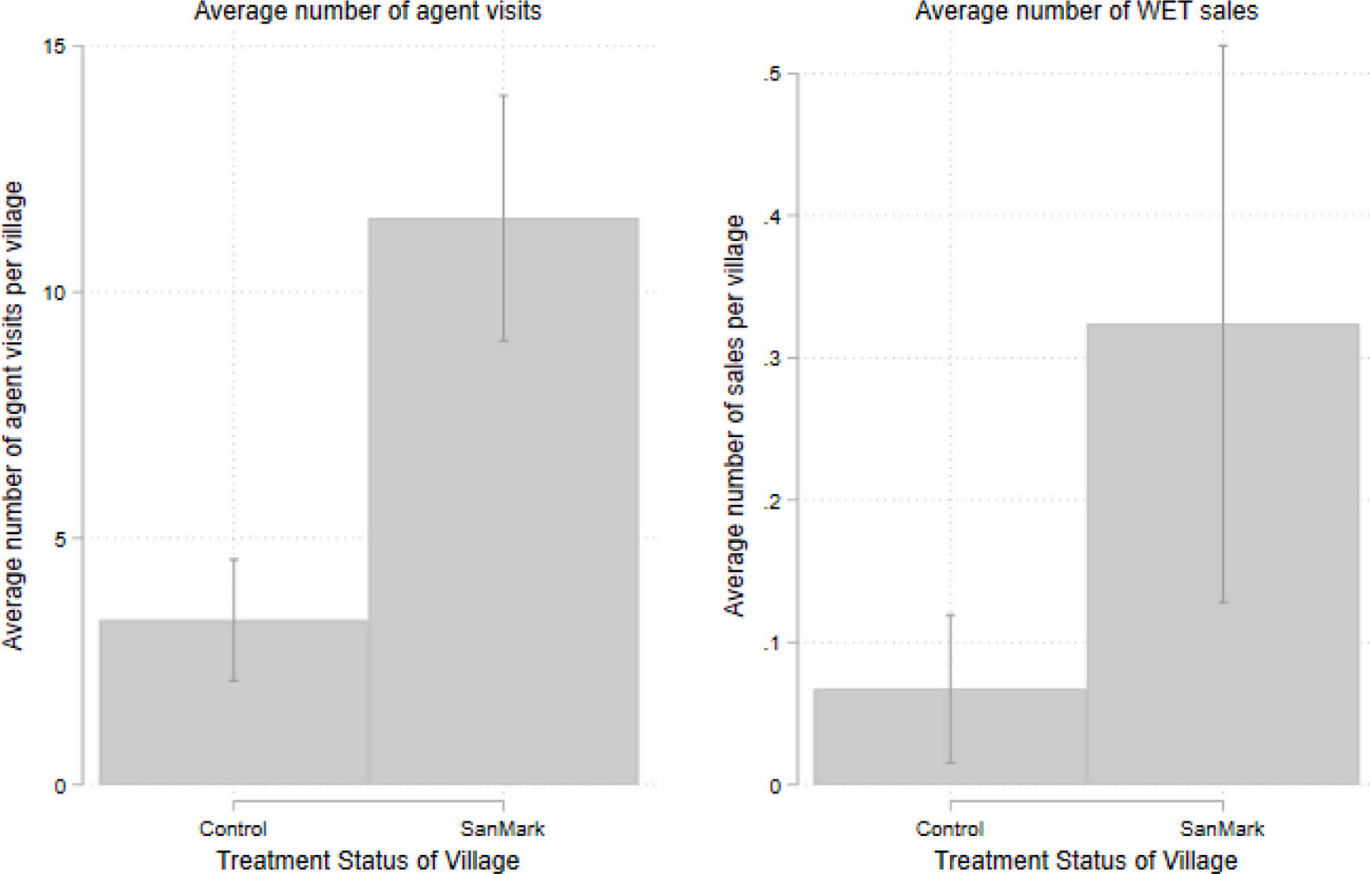
Average door-to-door agent sales and visits.*Note:* Agents were asked for the number of households visited and number of WET products sold in only the three main communities they visited. Hence, the total number of visits and sales in this figure underestimates the actual totals.

Sales agents sold close to 200 WET products over the whole study period, which is roughly half of all WET sales, as recorded by the internal administrative tracking data. Although this indicates that sales agents are an important driver of WET toilet uptake, the overall number of 400 WET products sold seems low. Most visits and sales conducted by sales agents were to households located in SanMark communities as defined by our randomized treatment allocation (see Appendix Fig. J.1). Overall, agents report to have made sales in 49% of treatment communities and in 17% of control communities.^18^ This suggests that spillovers may slightly attenuate SanMark impacts on toilet ownership but would not wipe them out.

## 6 Conclusion

This paper evaluates the impact of the SanMark intervention on sanitation coverage in Nigeria. The intervention was run as a randomized controlled trial that aimed to simultaneously stimulate demand for and supply of improved toilets by developing a new range of improved toilet products (known as WET models) that were designed with consumer preferences in mind, while being cheaper and more water efficient than others previously on the market. The intervention then engaged businesses to sell the WET products through demonstrations and business skills training, and mobilizing sales agents to promote the products to households by conducting door-to-door marketing and community activities.

Although the intervention holds promise in engaging the supply side, we find that the intervention was ineffective in shifting sanitation ownership rates and practices among households. This finding also holds for households that resided in SanMark communities, where sales agents were from and were more likely to be exposed to SanMark marketing activities *and* in which a treated business was located; and for households in communities that were exposed to CLTS 1.5 years before.

There are two important factors that may have contributed to SanMark not having had the desired impacts within the study time frame, which can provide lessons for future market-based sanitation programs.

First, although sales agents are likely to be pro-socially motivated, which theoretically reduces the need for external incentives (Besley & Ghatak, 2018), their financial returns from the activity were low. Despite being responsible for 50% of the total WET products sold, agents report limited sales activity. Informal discussions with agents suggest that this is due to the low incentives provided by the commission reimbursement system, which leads to reduced efforts to create demand. This suggests a need to adjust the incentive design. Aligning the business and sales agent training more closely could be an alternative approach.

Second, affordability challenges and liquidity constraints may have also played a role. Many households in the study area considered the WET models, although cheaper than other models, to be too expensive or reported other financial constraints. When households were asked why they do not plan to build or repair a toilet, 53% of respondents stated that they cannot afford it and a further 32% said that it is too expensive. This is also reflected by sales agents who were asked the most important reason why households decided **not** to buy a WET model: 87% mentioned some form of financial constraint. In line with this, Agarwal, Kohli, Chennuri, and Jenkins (2020) argue that consumer financing strategies are a key factor for success in market-based sanitation programs. Supporting evidence in the literature shows that both providing subsidies and offering sanitation credit can significantly increase sanitation uptake (see for example Augsburg et al., 2023, BenYishay et al., 2017, Deb et al., 2024, Guiteras et al., 2015, Lipscomb and Schechter, 2018, Lipscomb and Schechter, 2018, Pattanayak et al., 2009).

## CRediT authorship contribution statement

**Laura Abramovsky:** Writing – original draft, Validation, Supervision, Project administration, Methodology, Investigation, Funding acquisition, Formal analysis, Data curation, Conceptualization. **Nneka Akwunwa:** Writing – review & editing, Investigation. **Britta Augsburg:** Writing – review & editing, Writing – original draft, Validation, Supervision, Project administration, Methodology, Investigation, Funding acquisition, Formal analysis, Data curation, Conceptualization. **Ephraim Danladi:** Writing – review & editing, Project administration, Investigation. **Erik Harvey:** Writing – review & editing, Investigation, Funding acquisition, Conceptualization. **Emmanuel Iorkumbur:** Writing – review & editing, Project administration. **Julia Loh:** Writing – review & editing, Writing – original draft, Visualization, Investigation. **Melanie Lührmann:** Writing – original draft, Methodology, Investigation, Formal analysis. **Abdulazeez Musa:** Writing – review & editing, Project administration, Investigation, Conceptualization. **Ada Oko-Williams:** Writing – review & editing, Project administration, Funding acquisition, Conceptualization. **Harriet Olorenshaw:** Visualization, Investigation. **Francisco Oteiza:** Writing – original draft, Visualization, Validation, Project administration, Methodology, Investigation, Formal analysis, Data curation. **Juan Pablo Rud:** Writing – original draft, Supervision, Methodology, Investigation, Formal analysis. **Kyla Smith:** Writing – review & editing, Project administration, Investigation, Conceptualization.

## Declaration of competing interest

The authors declare that they have no known competing financial interests or personal relationships that could have appeared to influence the work reported in this paper.

## Figures and Tables

WaterAid Nigeria

WaterAid Nigeria

Own data, business and household census.

**Table D.1:** Definitions of control variables used in regressions.

Variables	Definition
Innovation metric	PCA of the following binary variables:
	In the last 12 months, the business made major changes in
	– Ways of running the business
	– Ways of assigning responsibilities to staff
	– Methods of relating with other businesses and government institutions
	– Marketing concepts or strategies
	In the last 12 months, staff received training for
	– Development and/or introduction of new products/services
	– Accounting, book keeping, or more general business skills
	In the last 12 months, the business introduced
	– new/significantly improved goods
	– new/significantly improved services
	– new/significantly improved methods for producing/supplying goods/services
	In the last 12 months, goods and services innovations were
	– new to the market
	– only new to the business
	In the last 12 months, the business
	– used billboards,posters and outdoor advertising
	– used face to face marketing for corporate or government customers

Relative wealth index	PCA of the following variables on HH assets:
	– Besides the house the respondent is living in, the household owns any house elsewhere
	– Acres of agricultural land the HH owns (both irrigated and unirrigated)
	– Acres of non-agricultural land the HH owns
	– Who owns the land (HH member, relative not living in the HH, collective ownership, others)
	– The total value of the land the HH owns

**Table E.1:** Balance between Treatment and Control Businesses at Baseline.

	All	Control			Treatment-Control	
	Obs.	Obs.	Mean	SD	Coeff.	*p*-value
**Business characteristics**						
Years in Existence	112	57	6.09	5.34	0.28	0.81
Number of Full-time Employees	129	69	4.26	3.51	1.32	0.06*
Registered with authorities (%)	129	69	72.46	45.00	2.54	0.75
Level of education of main owner						
No qualifications (%)	124	67	4.48	20.84	−4.48	0.08*
Primary education (%)	124	67	16.42	37.32	−0.63	0.92
Secondary education (%)	124	67	40.30	49.42	1.81	0.84
Tertiary education (%)	124	67	38.81	49.10	3.30	0.71
Households main customers (%)	129	69	65.22	47.98	9.78	0.23
Customers pickup products (%)	129	69	81.16	39.39	−1.16	0.87
Innovation Score	129	69	4.12	3.71	−0.48	0.43
Number of other CBP businesses within 5 km	129	69	6.90	3.33	−0.18	0.76
Involved in sanitation (%)	129	69	5.80	23.54	−4.13	0.21
**Infrastructure**						
Connected to power grid (%)	129	69	4.35	20.54	3.99	0.36
Owns electricity generator (%)	129	69	42.03	49.72	2.97	0.74
Improved water source (%)	129	69	2.90	16.90	−2.90	0.16
Business has internet connection (%)	129	69	2.90	16.90	0.43	0.89
Business uses cell phones (%)	129	69	71.01	45.70	−4.35	0.60
Owns reliable means of transport (%)	129	69	63.77	48.42	11.23	0.17
**Business performance**						
Typical real monthly profits (’000 ₦, 2009 prices)	129	69	138.24	520.53	−93.27	0.23
Ever received a loan (%)	129	69	37.68	48.81	−2.68	0.75
Sales increased in past year (%)	129	69	57.97	49.72	−2.97	0.74

*Note*: Data from baseline and second wave business surveys carried out before the intervention. If business is not surveyed in the baseline survey, data from the second wave survey is used. The variation in sample sizes across variables is due to missing responses for individual questions in the survey. Variables that fed into the innovation score are displayed in Appendix Table D.1. * p<0.10, ** p<0.05, *** p<0.01. Obs. stands for observations, SD for standard deviation, coeff. for coefficient.

**Table E.2:** Test for non-random attrition - firms .

	Firm closed in both post-treatment survey waves	Missing business performance outcomes
	(1)	(2)
SanMark	0.059	0.061
	(0.058)	(0.068)
Years in Existence ’	−0.008**	−0.001
	(0.004)	(0.007)
Number of Full-time Employees	0.004	0.001
	(0.008)	(0.010)
Registered with authorities	0.075	0.062
	(0.056)	(0.084)
Primary education (%)’	0.175	0.213
	(0.109)	(0.130)
Secondary education (%)’	0.071	0.149
	(0.090)	(0.111)
Tertiary education (%)’	0.144	0.247**
	(0.087)	(0.114)
Households main customers	−0.024	0.028
	(0.083)	(0.089)
Customers pickup products	0.012	−0.068
	(0.069)	(0.094)
Innovation Score	0.005	0.011
	(0.008)	(0.011)
Number of other CBP businesses within 5 km	−0.004	−0.003
	(0.012)	(0.014)
Connected to power grid	0.019	−0.013
	(0.164)	(0.167)
Owns electricity generator	0.004	−0.028
	(0.059)	(0.078)
Improved water source	−0.087	−0.104
	(0.113)	(0.134)
Business has internet connection	−0.075	−0.236**
	(0.081)	(0.118)
Business uses cell phones	0.106	0.076
	(0.068)	(0.088)
Have reliable means of transport (own)	0.039	0.009
	(0.063)	(0.077)
Typical real monthly profits (’000 ₦, 2009 prices)	-0.000**	-0.000***
	(0.000)	(0.000)
Ever received a loan	−0.046	−0.063
	(0.067)	(0.075)
Sales increased in past year	0.023	−0.035
	(0.063)	(0.077)
Constant	−0.148	−0.091
	(0.175)	(0.205)

Observations	129	129
Mean probability of outcome = 1	0.093	0.140

*Note*: Results from regressing baseline characteristics on two definitions of attrition: (1) Closure of firms in both post-treatment surveys; (2) Missing business performance outcomes. * p<0.10, ** p<0.05, *** p<0.01. Data from baseline and second wave business surveys carried out before the intervention. Standard errors clustered at business level. Both columns include LGA fixed effects.

**Table E.3:** Test for non-random attrition - households.

	Not surveyed at endline		Missing baseline and/or endline data on sanitation	
	(1)	(2)	(3)	(4)
SanMark	−0.015	−0.011	−0.016	−0.011
	(0.011)	(0.011)	(0.012)	(0.011)
HH head male		0.014		0.013
		(0.010)		(0.010)
HH head age (years)		−0.003***		−0.003***
		(0.000)		(0.000)
HH head employed		−0.065***		−0.065***
		(0.014)		(0.014)
HH head finished primary school		−0.017		−0.015
		(0.012)		(0.012)
Household size		−0.024***		−0.023***
		(0.002)		(0.002)
Household has at least 1 child below 6 y/o		0.014		0.012
		(0.010)		(0.010)
HH primary activity is farming		−0.037***		−0.040***
		(0.010)		(0.011)
Relative Wealth Index		−0.005**		−0.005**
		(0.002)		(0.002)
Distance to nearest LGA HQ (in km)		−0.001		−0.002
		(0.002)		(0.002)
Number of HHs within 5km		−0.000**		−0.000**
		(0.000)		(0.000)
Constant	0.141***	0.470***	0.148***	0.485***
	(0.008)	(0.038)	(0.008)	(0.038)

Number of HHs	5,639	5,639	5,639	5,639
Number of communities	293	293	293	293

*Note*: Results from regressing baseline characteristics on two definitions of attrition: (1) Not surveyed at endline; (2) Missing baseline and/or data on sanitation ownership. * p<0.10, ** p<0.05, *** p<0.01. Standard errors clustered at community level. All columns include LGA fixed effects. Missing values are imputed with the sample mean.

**Table F.1:** Firm awareness and offering of WET products and sanitation by SanMark treatment status (with controls)

	Firm knows about WET products	Firm offers WET products to consumers	Firm is involved in sanitation
	(1)	(2)	(3)
SanMark	0.10*	0.15**	0.24***
	(0.06)	(0.06)	(0.07)

Firm controls	Yes	Yes	Yes
Control mean at endline	0.85	0.03	0.08
No. of obs.	117	117	117

*Note:* Estimates based on OLS regressions using equation (1) with controls. All specifications include strata indicators (LGA). Standard errors are clustered at the business level and are shown in parentheses. * p<0.10, ** p<0.05, *** p<0.01. Dependent variable by columns: (1) *Firm knows about WET products*: an indicator variable equal to 1 if the business owner reports knowing about the WET product, 0 otherwise; (2) *Firm offers WET products to customers*: indicator variable equal to 1 if business owner reports offering WET products, 0 otherwise; (3) *Firm involved in sanitation:* indicator variable equal to 1 if business reports to offer WET products (as in Column 2) or having positive revenues from selling alternative toilet models, 0 otherwise. Firm controls included are: Number of full-time employees at baseline; educational attainment of main owner; indicator variable equal to 1 if educational attainment is imputed with mean value of sample due to missingness.

**Table F.2:** Firm involvement in sanitation by SanMark treatment status (ANCOVA).

	Firm involved in sanitation	
	(1)	(2)
SanMark	0.25***	0.25***
	(0.07)	(0.07)

Firm controls	No	Yes
Control mean at endline	0.08	0.08
No. of obs.	117	117

*Note:* Estimates based on ANCOVA specification, equation (3) with controls. All specifications include strata indicators (LGA). Standard errors are clustered at the business level and are shown in parentheses. * p<0.10, ** p<0.05, *** p<0.01. Dependent variable in both columns: *Firm involved in sanitation:* indicator variable equal to 1 if business reports to offer WET products (as in Column 2) or having positive revenues from selling alternative toilet models, 0 otherwise. Firm controls included are: Number of full-time employees at baseline; educational attainment of main owner.

**Table G.1:** Balance in baseline characteristics between treatment and control businesses.

	All	Control			Treatment-Control	
	Obs.	Obs.	Mean	SD	Coeff.	*p*-value
**Business performance**						
Typical real monthly profits (’000 ₦, 2009 prices)	111	62	153.92	547.34	−97.07	0.28
Typical real monthly sales (’000 ₦, 2009 prices)	111	62	161.58	556.89	−103.33	0.26
Typical real monthly costs (’000 ₦, 2009 prices)	111	62	7.66	35.84	−6.26	0.17
**Business performance - removing outliers**						
Typical real monthly profits (’000 ₦, 2009 prices)	91	49	25.62	179.21	−25.46	0.32
Typical real monthly sales (’000 ₦, 2009 prices)	91	48	34.01	221.02	−32.53	0.31
Typical real monthly costs (’000 ₦, 2009 prices)	91	47	1.75	1.57	−0.29	0.33

*Note*: Data from baseline and second wave business surveys carried out before the intervention. If business is not surveyed in the baseline survey, data from the second wave survey is used. The availability of the business for the baseline survey is not correlated to treatment status. The variation in sample sizes across variables is due to missing responses for individual questions in the survey. * p<0.10, ** p<0.05, *** p<0.01. Obs. stands for observations, SD for standard deviation, coeff. for coefficient. Outliers are defined as values above the 95th percentile or below the 5th percentile of the outcome of interest.

**Table G.2:** Balance between pre-intervention characteristics of firms with any missing data on business performance.

	All	Non-missing			Missing - non-missing	
	Obs.	Obs.	Mean	SD	Coeff.	*p*-value
SanMark	129	111	0.44	0.50	−0.17	0.18
**Business characteristics**						
Years in Existence	112	95	6.26	5.92	0.26	0.87
Number of Full-time Employees	129	111	4.86	4.16	−0.08	0.91
Registered with authorities (%)	129	111	72.07	45.07	−11.26	0.25
Primary education (%)	124	108	15.74	36.59	−3.01	0.77
Secondary education (%)	124	108	43.52	49.81	18.52	0.12
Tertiary education (%)	124	108	37.96	48.76	−18.29	0.17
Households main customers (%)	129	111	69.37	46.30	−2.85	0.80
Customers pickup products (%)	129	111	81.98	38.61	9.76	0.39
Innovation Score	129	111	3.81	3.46	−0.58	0.52
**Infrastructure**						
Connected to power grid (%)	129	111	5.41	22.71	−5.71	0.46
Owns electricity generator (%)	129	111	41.44	49.49	−14.11	0.27
Improved water source (%)	129	111	1.80	13.36	1.80	0.16
Business has internet connection (%)	129	111	3.60	18.72	3.60	0.04**
Business uses cell phones (%)	129	111	68.47	46.67	−3.75	0.74
Have reliable means of transport (own) (%)	129	111	67.57	47.02	−10.21	0.35
Ever received a loan (%)	129	111	36.04	48.23	−2.85	0.82
Sales increased in past year (%)	129	111	58.56	49.49	14.11	0.27
Number of other CBP businesses within 5 km	129	111	6.80	3.41	−0.09	0.91

*Note*: Data from baseline and second wave business surveys carried out before the intervention. ‘Non-missing‘ are observations where the business owner reported business performance outcomes, ‘Missing‘ refers to those where this information was not reported. Variables that fed into the innovation score are displayed in Appendix Table D.1. * p<0.10, ** p<0.05, *** p<0.01. Obs. stands for observations, SD for standard deviation, Coeff. for coefficient.

**Table G.3:** SanMark treatment status and business performance.

	IHS typical real monthly profits	Log typical real monthly sales	Log typical real monthly expenditures
	(1)	(2)	(3)
SanMark	−0.13	−0.27	−0.18
	(1.44)	(0.45)	(0.32)
SanMark × Post	1.08	0.45	0.17
	(1.94)	(0.49)	(0.31)

Control mean at endline	4.03	7.33	7.12
Control mean at endline (in levels)	886.80	2904.89	2018.10
No. of obs.	111	111	111

*Note*: Estimates based on OLS regressions using equation (3). *p*-values from individual testing are presented in brackets. All specifications include strata indicators (LGAs). Standard errors are clustered at the business level and are shown in parenthesis * p<0.10, ** p<0.05, *** p<0.01. Dependent variable by columns: (1) *IHS typical monthly profit margin*: inverse hyperbolic sine (IHS) of monthly profits calculated from self-reported monthly sales and expenditures; (2) *Log typical monthly sales*: the natural logarithm of self-reported monthly sales; (3) *Log typical monthly expenditures:* the natural logarithm of self-reported monthly expenditures. The dependent variables are presented in terms of 2009 prices.

**Table G.4:** SanMark treatment status and business performance - removing outliers.

	IHS typical real monthly profits	Log typical real monthly sales	Log typical real monthly expenditures
	(1)	(2)	(3)
SanMark	0.19	−0.07	−0.14
	(1.46)	(0.31)	(0.25)
SanMark × Post	0.47	0.28	−0.05
	(1.90)	(0.34)	(0.26)

Control mean at endline	4.23	7.29	7.32
Control mean at endline (in levels)	601.84	2254.14	1870.59
No. of obs.	91	91	91

*Note*: Estimates based on OLS regressions using equation (3). Outliers, defined as values above the 95th percentile or below the 5th percentile, are removed. *p*-values from individual testing are presented in brackets. All specifications include strata indicators (LGAs). Standard errors are clustered at the business level and are shown in parenthesis * p<0.10, ** p<0.05, *** p<0.01. Dependent variable by columns: (1) *IHS typical monthly profit margin*: inverse hyperbolic sine (IHS) of monthly profits calculated from self-reported monthly sales and pre-tax expenditures; (2) *Log typical monthly sales*: the natural logarithm of self-reported monthly sales; (3) *Log typical monthly expenditures:* the natural logarithm of self-reported monthly expenditures. The dependent variables are presented in terms of 2009 prices.

**Table G.5:** SanMark treatment status and business performance (with DiD and ANCOVA)

	IHS typical monthly profit margin		Log typical monthly sales		Log typical monthly pre-tax exp	
	(1)	(2)	(3)	(4)	(5)	(6)
SanMark	−0.21	0.46	−0.42	0.09	−0.34	0.01
	(1.40)	(0.94)	(0.46)	(0.23)	(0.33)	(0.18)
SanMark × Post	0.81		0.49		0.31	
	(1.93)		(0.53)		(0.32)	

ANCOVA	No	Yes	No	Yes	No	Yes
Firm controls	Yes	Yes	Yes	Yes	Yes	Yes
Control mean at endline	4.03	4.03	7.33	7.33	7.12	7.12
No. of obs.	111	111	111	111	111	111

*Note*: Estimates based on OLS regressions using equation (3). All specifications include strata indicators (LGS). Standard errors are clustered at the business level and are shown in parentheses * p<0.10, ** p<0.05, *** p<0.01. Dependent variable by columns: (1) and (2) *IHS typical monthly profit margin*: inverse hyperbolic sine (IHS) of monthly profits calculated from self-reported monthly sales and pre-tax expenditures; (3) and (4) *Log typical monthly sales*: the natural logarithm of self-reported monthly sales; (5) and (6) *Log typical monthly expenditures:* the natural logarithm of self-reported monthly expenditures, pre-tax. Firm controls included are: Number of full-time employees at baseline; educational attainment of main owner; indicator variable equal to 1 if educational attainment is imputed with mean value of sample due to missingness.

**Table G.6:** Firm awareness and offering of WET products and sanitation by SanMark treatment status (selected sample with controls).

	Firm knows about WET products	Firm offers WET products to consumers	Firm is involved in sanitation
	(1)	(2)	(3)
SanMark	0.15***	0.16**	0.21***
	(0.05)	(0.06)	(0.07)

Firm controls	Yes	Yes	Yes
Control mean at endline	0.85	0.03	0.06
No. of obs.	111	111	111

*Note:* Estimates based on OLS regressions using equation (1). Sample is reduced to those businesses that reported business performance outcomes. Standard errors are clustered at the business level and are shown in parentheses. * p<0.10, ** p<0.05, *** p<0.01. All specifications include strata indicators (LGA). Dependent variable by columns: (1) *Firm knows about WET products*: an indicator variable equal to 1 if the business owner reports knowing about the WET product, 0 otherwise; (2) *Firm offers WET products to customers*: indicator variable equal to 1 if business owner reports offering WET products, 0 otherwise; (3) *Firm involved in sanitation:* indicator variable equal to 1 if business reports to offer WET products (as in Column 2) or having positive revenues from selling alternative toilet models, 0 otherwise. Firm controls included are: Number of full-time employees at baseline; educational attainment of main owner; indicator variable equal to 1 if educational attainment is imputed with mean value of sample due to missingness.

**Table H.1:** Impact of SanMark on toilet ownership.

	HH owns a functioning toilet		HH owns a functioning improved toilet	
	(1)	(2)	(3)	(4)
SanMark	−0.01	−0.02	−0.01	−0.02
	(0.02)	(0.02)	(0.02)	(0.02)
SanMark × Post	−0.01		−0.02	
	(0.02)		(0.02)	

ANCOVA	No	Yes	No	Yes
Controls	Yes	Yes	Yes	Yes
Control mean at endline	0.54	0.54	0.49	0.49
No. of communities	291	291	291	291
No. of HHs	4,703	4,703	4,703	4,703

*Note:* Table displays the results of the DiD and ANCOVA specifications for the impact of SanMark treatment on the outcome variables. Standard errors are clustered at the community level. Dependent variable by columns: (1) *HH owns functioning toilet*: an indicator variable equal to 1 if the household reports to own a functioning toilet, 0 otherwise; (2) *HH owns a functioning improved toilet*: indicator variable equal to 1 if household (HH) reports to own a functioning toilet that is improved as defined in WHO/UNICEF (2015). Controls: age, age squared, gender, education attainment level and employment status of the household head; household size, wealth asset score, and a dummy variable equal to one if farming is the household’s main economic activity. Standard errors are clustered at the community level. Standard errors are shown in parentheses * p<0.10, ** p<0.05, *** p<0.01.

**Table H.2:** Impact of SanMark on toilet ownership by CLTS treatment group - excluding pre-CLTS.

	HH owns a functioning toilet	HH owns a functioning improved toilet
SanMark × Post ($\tau_{2}$)	−0.04	−0.05
	(0.04)	(0.03)
SanMark × Post × CLTS-treated ($\tau_{3}$)	0.03	0.03
	(0.06)	(0.05)
Joint effect for SanMark and CLTS-treated ($\tau_{2}+\tau_{3}$)	−0.01	−0.02
	(0.04)	(0.04)

Control mean at endline	0.54	0.49
No. of communities	213	213
No. of HHs	3,429	3,429

*Note:* Estimates based on DiD specification using equation (5). All specifications include strata indicators (LGAs). Standard errors are clustered at the community level and are shown in parentheses * p<0.10, ** p<0.05, *** p<0.01. Dependent variable by columns: (1) *HH owns functioning toilet*: an indicator variable equal to 1 if the household reports to own a functioning toilet, 0 otherwise; (2) *HH owns a functioning improved toilet*: indicator variable equal to 1 if household (HH) reports to own a functioning toilet that is improved as defined in WHO/UNICEF (2015).

**Table H.3:** Impact of SanMark on toilet ownership by CLTS treatment group - with controls and ANCOVA.

	HH owns a functioning toilet		HH owns a functioning improved toilet	
	(1)	(2)	(3)	(4)
SanMark × Post ($\tau_{2}$)	−0.04	−0.04	−0.05	−0.04
	(0.04)	(0.03)	(0.03)	(0.03)
SanMark × Post × CLTS-treated ($\tau_{3}$)	0.04	0.03	0.05	0.03
	(0.05)	(0.04)	(0.04)	(0.04)
Joint effect for SanMark and CLTS-treated ($\tau_{2}+\tau_{3}$)	0.00	−0.01	0.00	−0.01
	(0.03)	(0.03)	(0.03)	(0.02)

ANCOVA	No	Yes	No	Yes
Controls	Yes	Yes	Yes	Yes
Control mean at endline	0.54	0.54	0.49	0.49
No. of communities	291	291	291	291
No. of HHs	4,703	4,703	4,703	4,703

*Note:* Table displays the results of the DiD and ANCOVA specifications for the impact of SanMark treatment on the outcome variables by CLTS treatment status. The relevant variable of interest is SanMark × Post for the DiD specification and SanMark for the ANCOVA specification. Dependent variable by columns: (1) *HH owns functioning toilet*: an indicator variable equal to 1 if the household reports to own a functioning toilet, 0 otherwise; (2) *HH owns a functioning improved toilet*: indicator variable equal to 1 if household (HH) reports to own a functioning toilet that is improved as defined in WHO/UNICEF (2015). Controls: age, age squared, gender, education attainment level and employment status of the household head; household size, wealth asset score, and a dummy variable equal to one if farming is the household’s main economic activity. Standard errors are clustered at the community level and are shown in parentheses * p<0.10, ** p<0.05, *** p<0.01.

**Table H.4:** Impact of SanMark on toilet ownership by CLTS treatment group - with controls and ANCOVA (excluding pre-CLTS).

	HH owns a functioning toilet		HH owns a functioning improved toilet	
	(1)	(2)	(3)	(4)
SanMark × Post ($\tau_{2}$)	−0.04	−0.03	−0.05	−0.04
	(0.04)	(0.03)	(0.03)	(0.03)
SanMark × Post × CLTS-treated ($\tau_{3}$)	0.03	0.01	0.03	0.01
	(0.06)	(0.05)	(0.05)	(0.05)
Joint effect for SanMark and CLTS-treated ($\tau_{2}+\tau_{3}$)	−0.01	−0.02	−0.02	−0.04
	(0.04)	(0.03)	(0.04)	(0.03)

ANCOVA	No	Yes	No	Yes
Controls	Yes	Yes	Yes	Yes
Control mean at endline	0.54	0.54	0.49	0.49
No. of communities	213	213	213	213
No. of HHs	3,429	3,429	3,429	3,429

*Note:* Table displays the results of the DiD and ANCOVA specifications for the impact of SanMark treatment on the outcome variables by CLTS treatment status. The relevant variable of interest is SanMark × Post for the DiD specification and SanMark for the ANCOVA specification. Dependent variable by columns: (1) *HH owns functioning toilet*: an indicator variable equal to 1 if the household reports to own a functioning toilet, 0 otherwise; (2) *HH owns a functioning improved toilet*: indicator variable equal to 1 if household (HH) reports to own a functioning toilet that is improved as defined in WHO/UNICEF (2015). Controls: age, age squared, gender, education attainment level and employment status of the household head; household size, wealth asset score, and a dummy variable equal to one if farming is the household’s main economic activity. Standard errors are clustered at the community level and are shown in parentheses * p<0.10, ** p<0.05, *** p<0.01.

**Table H.5:** Balance between Treatment and Control households and communities (sample for Table 7) at Baseline.

	All	Control			Treatment-Control	
	Obs.	Obs.	Mean	SD	Coeff.	p-value
**Panel A - Household Characteristics**						
HH head male (%)	2926	1443	61.47	48.68	1.78	0.42
HH head age (years)	2926	1443	56.15	17.64	−0.22	0.80
HH head employed (%)	2926	1443	75.19	43.21	3.97	0.07*
HH head finished primary school (%)	2926	1443	66.81	47.11	−0.59	0.82
Household size	2926	1443	4.02	2.40	0.00	1.00
Household has at least 1 child below 6 y/o (%)	2926	1443	27.58	44.71	1.08	0.58
HH primary activity is farming (%)	2926	1443	48.16	49.98	1.80	0.69
Relative asset wealth index score (mean = 0, SD = 2)	2926	1443	−0.04	2.11	0.18	0.27
**Panel B - Toilet Ownership**						
Own a functioning toilet (any type) (%)	2926	1443	35.62	47.90	1.67	0.66
Own a functioning, improved toilet (%)	2926	1443	32.50	46.85	1.21	0.75
**Panel C - Community Characteristics**						
Distance to nearest LGA HQ (in km)	185	93	7.68	3.97	−1.24	0.04**
Number of households within a 5km radius	185	93	1413.76	990.24	198.87	0.17

*Note*: Data from community survey during wave 1 data collection and wave 2 household survey carried out before the intervention. Variables that fed into the asset wealth index are displayed in Appendix Table D.1. * p<0.10, ** p<0.05, *** p<0.01. Obs. stands for observations, SD for standard deviation, Coeff. for coefficient, HQ for head quarter, and BL for baseline.

**Table H.6:** Impact of SanMark on toilet ownership by treatment intensity.

	HH owns a functioning toilet		HH owns a functioning improved toilet	
	(1)	(2)	(3)	(4)
SanMark (× Post) × Treated Main Supplier	0.04	0.03	0.01	0.01
	(0.04)	(0.04)	(0.04)	(0.03)
SanMark (× Post) Control Main Supplier	−0.04	−0.03	−0.04	−0.04
	(0.03)	(0.03)	(0.03)	(0.03)
Difference	0.08	0.06	0.05	0.05
	(0.05)	(0.05)	(0.05)	(0.04)

ANCOVA	No	Yes	No	Yes
Controls	Yes	Yes	Yes	Yes
Control mean at endline	0.56	0.56	0.53	0.53
No. of communities	249	249	249	249
No. of HHs	4,022	4,022	4,022	4,022

*Note*: Heterogeneous treatment effects by treatment of main village concrete block supplier. The relevant variable of interest is SanMark × Post for the DiD specification and SanMark for the ANCOVA specification. Dependent variable by columns: (1) *HH owns functioning toilet*: an indicator variable equal to 1 if the household reports to own a functioning toilet, 0 otherwise; (2) *HH owns a functioning improved toilet*: indicator variable equal to 1 if household (HH) reports to own a functioning toilet that is improved as defined in WHO/UNICEF (2015). Household controls: age, age squared, gender, education attainment level and employment status of the household head, household size, wealth asset score, and a dummy variable equal to one if farming is the household’s main economic activity. Households with both control and treated main suppliers are dropped. Standard errors are clustered at the community level and are shown in parentheses * p<0.10, ** p<0.05, *** p<0.01.

**Table H.7:** Impact of SanMark on toilet ownership by treatment intensity full sample.

	(1)	(2)	(3)	(4)
	Nr of SanMark suppliers within …		Nr of SanMark suppliers within …	
	…1km	…5km	…1km	…5km
SanMark × Post	−0.01	−0.02	−0.02	−0.03
	(0.03)	(0.04)	(0.02)	(0.03)
SanMark × Post × Treatment intensity	−0.00	0.01	0.00	0.01
	(0.04)	(0.01)	(0.04)	(0.01)
Treatment intensity	0.11	0.06	0.09	0.06
	(0.04)	(0.01)	(0.04)	(0.01)

Control mean at endline	0.52	0.41	0.47	0.32
No. of communities	267	267	267	267
No. of HHs	4,445	4,445	4,445	4,445

*Note*: Estimates based on DiD specification using equation (5) of the main paper. All specifications include strata indicators (LGAs). Standard errors are clustered at the community level and are shown in parentheses * p<0.10, ** p<0.05, *** p<0.01. Dependent variable by columns: (1) and (2) *HH owns functioning toilet*: an indicator variable equal to 1 if the household reports to own a functioning toilet, 0 otherwise; (3) and (4) *HH owns a functioning improved toilet*: indicator variable equal to 1 if household (HH) reports to own a functioning toilet that is improved as defined in WHO/UNICEF (2015). Number of SanMark suppliers within a radius of distance defined by the number of SanMark suppliers within a radius of distance from the centroid of the community polygon.

## Data Availability

Data and replication files are included in this submission.

## Appendix A Study location

See Fig. A.1.

## Appendix B The WET models

See Fig. B.1, Fig. B.2.

## Appendix C Business networks

See Fig. C.1.

## Appendix D Indices

See Table D.1.

## Appendix E Attrition

### E.1 Firms

See Table E.1, Table E.2.

### E.2 Households

See Table E.3.

## Appendix F Alternative specifications - businesses

See Table F.1, Table F.2.

## Appendix G Business performance outcomes

We provide here a discussion of intervention impacts on business performance.

We start by providing information on the reported typical monthly sales and costs of businesses in Table G.1. Sales were ₦161,580 on average (approximately US$ 449 at the prevailing exchange rate during November 2017 of ₦360 = US$1.). With average reported costs on ₦7660, the typical business would make a monthly profit of ₦146,780.

Given that a significant proportion of businesses (14%) do not report business performance outcomes (see Panel B of Table 2), we investigate whether our sample remains balanced when taking into account item non-response. We show that treatment does not predict business performance item non-response in Table G.2, even after controlling for business characteristics (see Table E.2). The missing–non-missing comparison in the last columns of Table G.2 shows that there are no significant differences between businesses with missing and non-missing business performance outcomes at baseline, except for a businesses with missing business performance outcomes being 3.6 percentage points more likely to have Internet connection at baseline.

We next turn to estimating impacts on these business performance outcomes. We use the following specification:

(5) $$y_{i,g,t}=\alpha +\nu SanMark_{i}^{b}+\tau SanMark_{i}^{b}\times Post_{t}+X_{i}^{\prime }\beta +Post_{t}+\mu_{g}+\epsilon_{i,g,t}$$

where $y_{i,g,t}$ represents the business outcome of interest for business i in LGA g at period t. $SanMark_{i}^{b}$ is an indicator variable equal to 1 if business i was selected into the SanMark programme, 0 otherwise. $Post_{t}$ is an indicator equal to 1 if the period of analysis t is after the introduction of SanMark (in waves 3 and 4) and 0 otherwise (in wave 2). Here, τ is our coefficient of interest, as it represents the difference in outcomes for the SanMark businesses before and after the intervention. $X_{i}^{\prime }$ is a vector of control variables at the business level, while $Post_{t}$ allows for a common time trend. $\mu_{g}$ controls for time-invariant differences in the outcomes across LGAs, given that our randomization was stratified at that level. This specification pools all post-SanMark observations together (from both waves 3 and 4) to improve precision.

Results are presented in Table G.3. Given significant outliers we transform the outcomes (natural logarithms for sales and costs and inverse hyperbolic spine for profits given significant number of negative profits). We find that, while estimated coefficients are positive, they are statistically insignificant. When removing outliers the coefficients remain positive and insignificant but become significantly smaller in magnitude (Table G.4). Results are also consistent when using an ANCOVA specification with and without covariates (Table G.5).

For completeness, we also show technology adoption impacts on the sample of 111 businesses in Table G.6.

## Appendix H Alternative specifications - households

### H.1 SanMark impact on toilet ownership - robustness

See Table H.1.

### H.2 Heterogeneous impacts by CLTS treatment group - robustness

See Table H.2, Table H.3, Table H.4.

### H.3 Balance table - selected sample of households

See Table H.5.

### H.4 Heterogeneous impacts by SanMark treatment intensity - robustness

See Table H.6, Table H.7.

## Appendix I Power analysis

We conduct a power analysis for this study using information on sample size, baseline mean, and intra-cluster correlation (ICC). We focus on ownership of a functioning and improved toilet (figures are similar for ownership of a functioning toilet). We calculate minimum detectable effects (MDEs) for a power of 0.8 and a significance level of 0.05, assuming two-tailed t-tests for our binary outcome variable. The raw ICC is 0.2039, yielding an MDE of 0.0964. Factoring in household size and information on the household head (gender, age, and whether employed or not) the ICC drops to 0.063, resulting in an MDE of 0.0589. This implies that our sample possesses the capability to detect a reduction of just under six percentage points with statistical significance.

Our estimated coefficient is −0.02 with a standard error of 0.021, implying a range for true effect sizes spanning from −0.061 to 0.021.

## Appendix J Sales agent activities

See Fig. J.1.

## Footnotes

1 See for example United Nations Deputy Secretary-General Jan Eliasson’s call for action in 2013 https://news.un.org/en/story/2013/03/435102.

2 The term was first formally defined in Devine and Kullmann (2011).

3 The approach has been implemented in several countries, including Cambodia, Vietnam, Bangladesh, Tanzania, Nigeria, and Peru. Most evidence on the effectiveness of this intervention so far is descriptive Hoo et al. (2022) and USAID (2018) being one exception.

4 Depending on the model, prices were reduced by 20%–80% compared with the median toilet cost reported in our sample. The significant barrier that such high costs may pose for sanitation investments was recently highlighted by Voth-Gaeddert, Fikru, and Oerther (2022).

5 Zuin et al. (2019) discuss the widespread adoption of CLTS in the rural sanitation sector.

6 We discuss the evaluation of the CLTS component of this program in some detail in the next section. Abramovsky, Augsburg, Lührmann, Oteiza, and Rud (2023) present the results of the full impact evaluation.

7 In line with this, Peletz, Cock-Esteb, Ysenburg, Haji, Khush, and Dupas (2017) and Peletz et al. (2019) show that households’ willingness to pay for latrines is below market prices in Tanzania and Kenya, respectively, with a lack of cash cited as the key underlying reason.

8 LGAs are Nigeria’s second administrative subdivision, led by a local government council.

9 At the time of the study, the 2014 National Nutrition and Health Survey showed that 29% of households in Enugu and 46% of households in Ekiti had access to improved sanitation. Open defecation rates were 51% and 44% respectively.

10 American Standard SaTo pans are colorful plastic pans that reduce the need for water to flush and mechanically seal pit latrines.

11 These activities were initially conducted by LGAs’ WASH unit staff and, later on, by the sales agents and local businesses.

12 One way of avoiding such spillover effects would have been to randomize local markets to treatment and control, rather than individual firms. However, there would not have been a sufficient number of separate and isolated markets in our sample to do this.

13 We also checked that pre-treatment characteristics are balanced across treatment and control groups for the full baseline sample and find the same results (Appendix Table E.1).

14 The corresponding balance table of baseline characteristics for this selected sample is presented in Table 2.

15 The balance in baseline characteristics between treatment and control households are presented in Table 3.

16 Note that we were just not powered to detect such small impacts, and hence we cannot be certain whether this is a tightly estimated null — essentially a ‘true zero’ — or a potentially noisy, very small, and negative effect. The original study, while assuming two-sided tests, focused on positive minimum detectable effects and powered the study to detect impacts of just under 10 ppts. Considering that the aim of the intervention was to elevate communities from an average sanitation coverage of 36% to complete coverage, this seemed a conservative enough approach. In Appendix I, we present an updated power analysis showing that we are powered to detect significantly smaller impacts (ranging from −0.057 to 0.037 for ownership of a functioning toilet, and −0.061 to 0.027 for ownership of a functioning improved toilet), though not small enough to establish a tightly estimated null.

17 The balance in baseline characteristics between treatment and control households for this selected sample are presented in Appendix Table H.5.

18 We asked sales agents to identify the three main communities they performed household visits and WET sales in. This could include, of course, the agent’s own community, which by definition would have been a SanMark treatment TU. When these communities fell within our study area, we then matched these TUs with their corresponding SanMark treatment status.

## References

[b1] Abramovsky L., Augsburg B., Lührmann M., Oteiza F., Rud J.P. (2023). Community matters: Heterogeneous impacts of a sanitation intervention. World Development.

[b2] Abramovsky L., Augsburg B., Oteiza F. (2019).

[b3] Adukia A. (2017). Sanitation and education. American Economic Journal: Applied Economics.

[b4] Agarwal R., Kohli A., Chennuri S., Jenkins M.W. (2020). Global assessment of grant-funded, market-based sanitation development projects. Waterlines.

[b5] Augsburg B., Caeyers B., Giunti S., Malde B., Smets S. (2023). Labeled loans and human capital investments. Journal of Development Economics.

[b6] Augsburg B., Foster A., Johnson T., Lipscomb M. (2024). Evidence on designing sanitation interventions. Journal of Development Economics.

[b7] Augsburg B., Rodriguez-Lesmes P. (2018). Sanitation and child health in India. World Development.

[b8] BenYishay A., Fraker A., Guiteras R., Palloni G., Shah N., Shirrell S. (2017). Microcredit and willingness to pay for environmental quality: Evidence from a randomized-Controlled trial of finance for sanitation in rural cambodia. Journal of Environmental Economics and Management.

[b9] Besley T., Ghatak M. (2018). Prosocial motivation and incentives. Annual Review of Economics.

[b10] Cameron L., Gertler P., Shah M., Alzua M.L., Martinez S., Patil S. (2022). The dirty business of eliminating open defecation: The effect of village sanitation on child height from field experiments in four countries. Journal of Development Economics.

[b11] de Mel S., McKenzie D., Woodruff C. (2009). Measuring microenterprise profits: Must we ask how the sausage is made?. Journal of Development Economics.

[b12] Deb S., Joseph G., Andres L.A., Grabinsky Zabludovsky J. (2024). Is the glass half full or half empty? Examining the impact of Swatch Bharat interventions on sanitation and hygiene in rural Punjab, India. Journal of Development Economics.

[b13] Deutschmann J.W., Gars J., Houde J.-F., Lipscomb M., Schechter L. (2023). Privatization of public goods: evidence from the sanitation sector in senegal. Journal of Development Economics.

[b14] Devine J., Kullmann C. (2011).

[b15] FMWR/NBS/UNICEF (2022).

[b16] Galiani, S. (2021). *Public sector participation in the water sector: Opportunities and pitfalls*: *Tech. rep.*, Available at SSRN.

[b17] Galiani S., Gertler P., Schargrodsky E. (2005). Water for life: The impact of the privatization of water services on child mortality. Journal of Political Economy.

[b18] Guiteras R., Levinsohn J., Mobarak A. (2015). Encouraging sanitation investment in the developing world: A cluster-randomized trial. Science.

[b19] Hoo Y.R., Joseph G., Rivera R., Smets S., Nguyen H., Ljung P. (2022). Strategic complements: Poverty-targeted subsidy programs show additive benefits on household toilet purchases in rural Cambodia when coupled with sanitation marketing. PLoS ONE.

[b20] Houde J.-F., Johnson T., Lipscomb M., Schechter L. (2022).

[b21] Lipscomb M., Schechter L. (2018). Subsidies versus mental accounting nudges: Harnessing mobile payment systems to improve sanitation. Journal of Development Economics.

[b22] Orgill-Meyer J., Pattanayak S. (2020). Improved sanitation increases long-term cognitive test scores. World Development.

[b23] Pattanayak S.K., Yang J.-C., Dickinson K.L., Poulos C., Patil S.R., Mallick R.K. (2009). Shame or subsidy revisited: social mobilization for sanitation in Orissa, India. Bulletin of the World Health Organization.

[b24] Peletz R., Cock-Esteb A., Ysenburg D., Haji S., Khush R., Dupas P. (2017). Supply and demand for improved sanitation: Results from randomized pricing experiments in Rural Tanzania. Environmental Science and Technology.

[b25] Peletz R., Kisiangani J., Ronoh P., Cock-Esteb A., Chase C., Khush R. (2019). Assessing the demand for plastic latrine slabs in rural Kenya. American Journal of Tropical Medicine and Hygiene.

[b26] Sahoo K., Hulland K., Caruso B., Swain R., Freeman M., Panigrahi P. (2015). Sanitation-related psychosocial stress: A grounded theory study of women across the life-course in Odisha, India. Social Science & Medicine.

[b27] USAID (2018).

[b28] Voth-Gaeddert L.E., Fikru M.G., Oerther D.B. (2022). Limited benefits and high costs are associated with low monetary returns for Guatemalan household investment in water, sanitation, and hygiene technologies. World Development.

[b29] WaterAid (2018).

[b30] WHO/UNICEF (2015).

[b31] WHO/UNICEF (2023).

[b32] WSP (2011). *Economic impacts of inadequate sanitation in India*: *Tech. rep.*.

[b33] Zuin V., Delaire C., Peletz R., Cock-Esteb A., Khush R., Albert J. (2019). Policy diffusion in the rural sanitation sector: Lessons from community-led total sanitation (CLTS). World Development.

